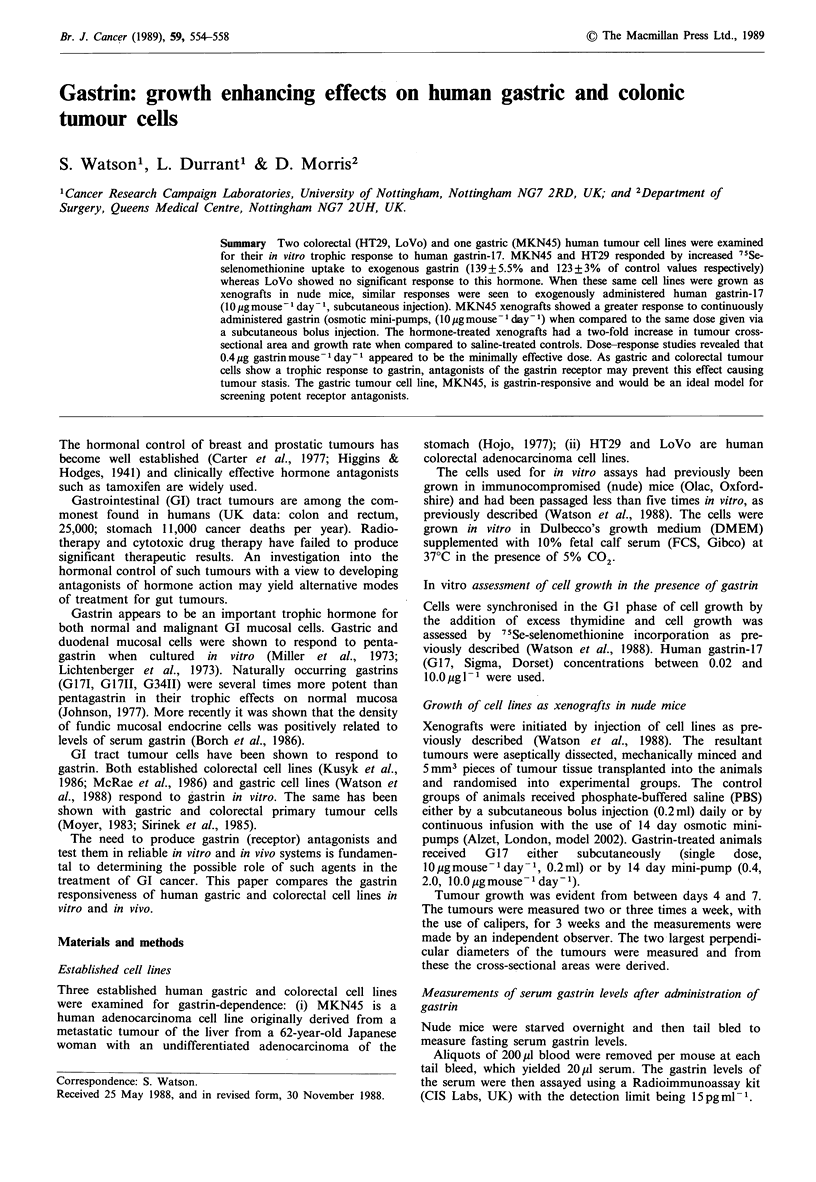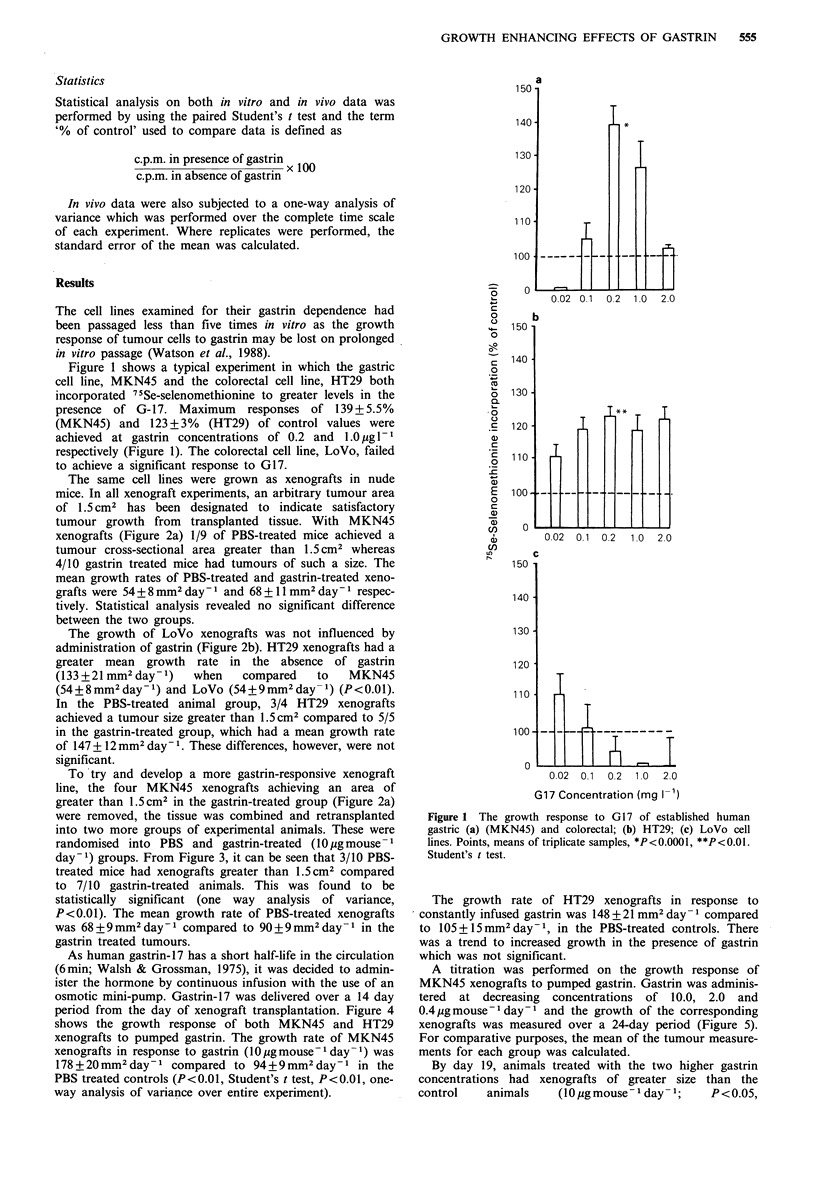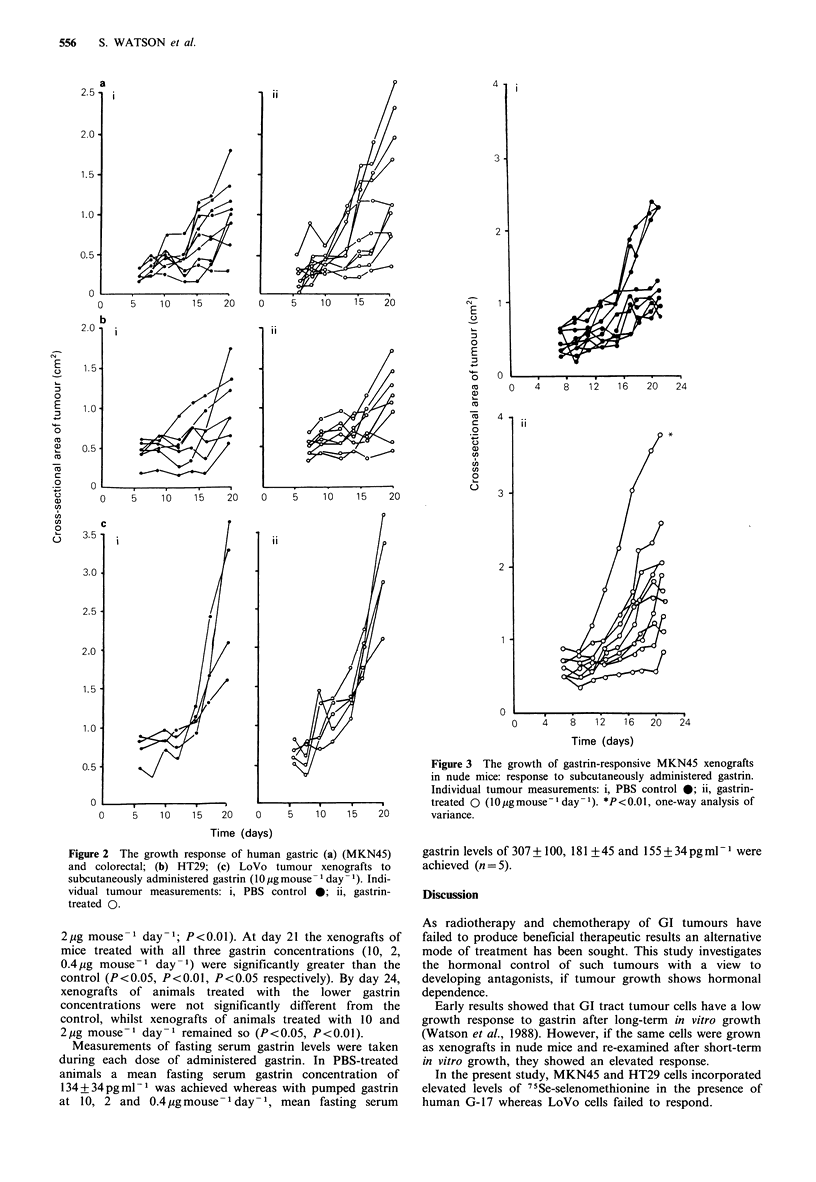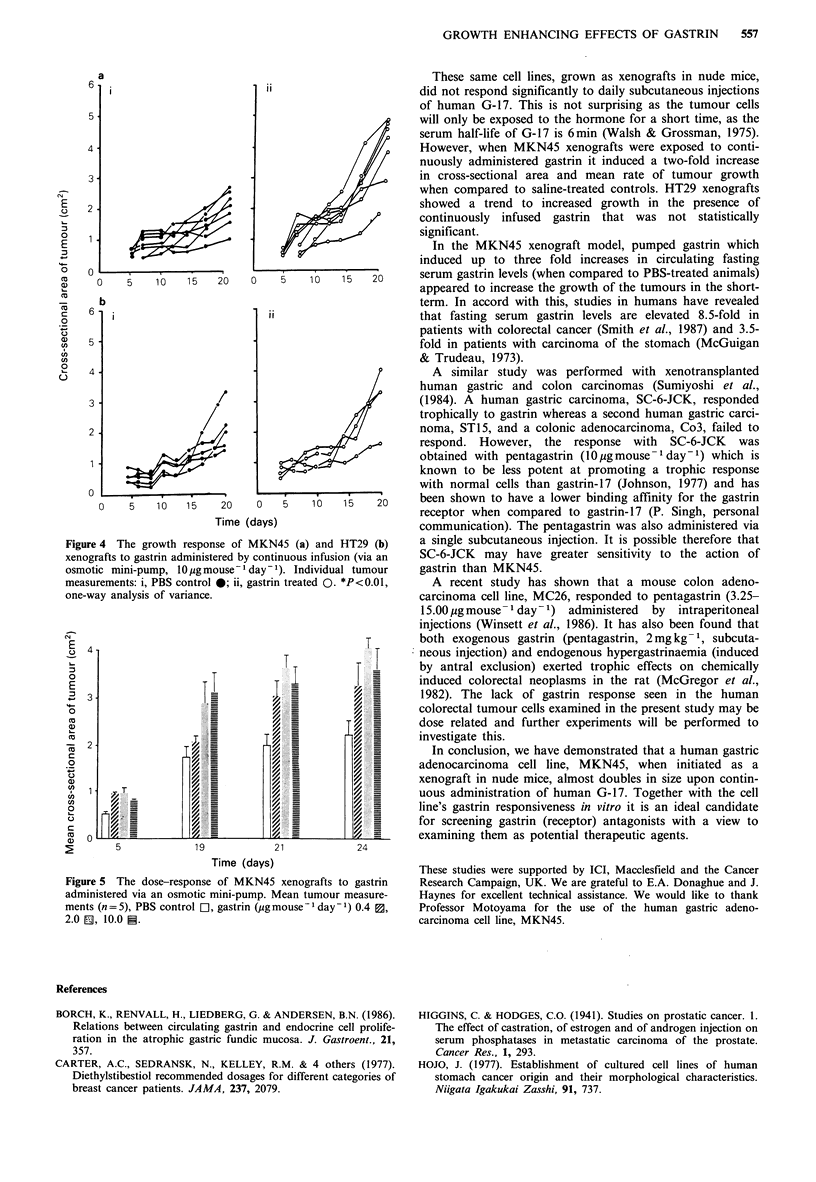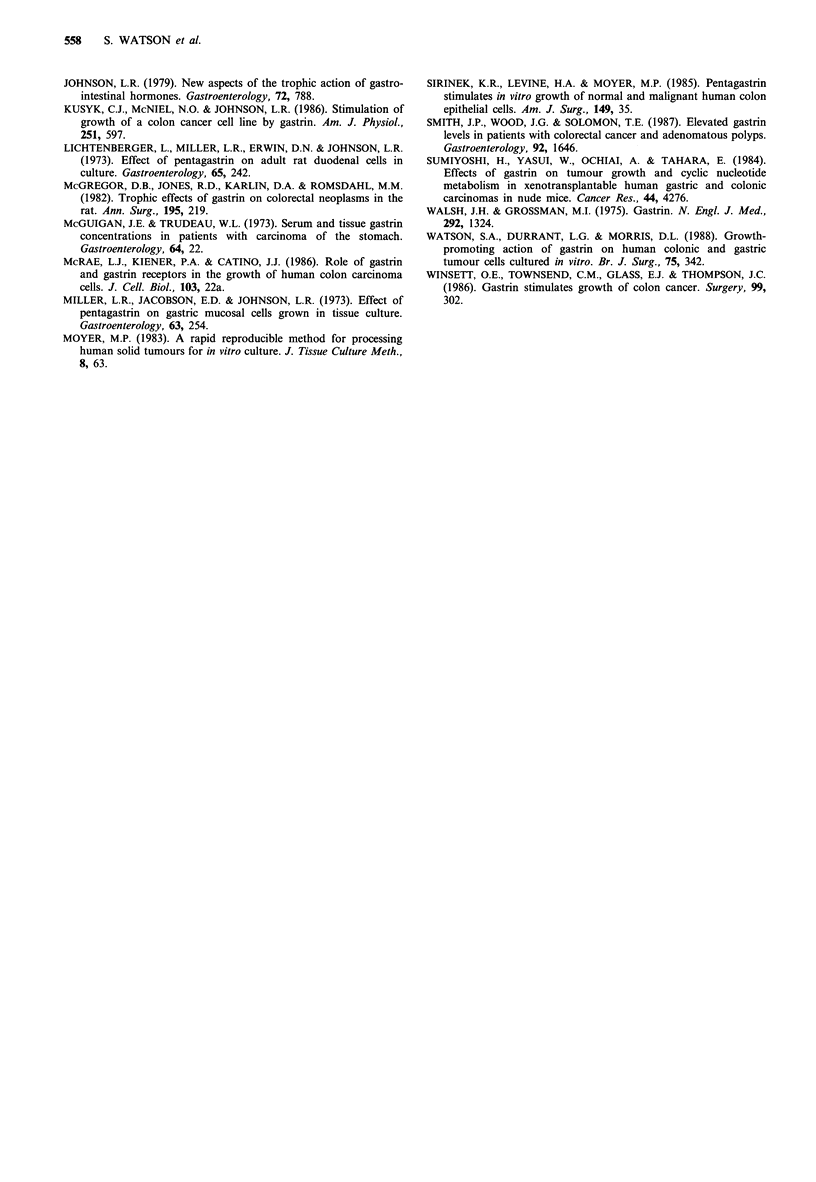# Gastrin: growth enhancing effects on human gastric and colonic tumour cells.

**DOI:** 10.1038/bjc.1989.112

**Published:** 1989-04

**Authors:** S. Watson, L. Durrant, D. Morris

**Affiliations:** Cancer Research Campaign Laboratories, University of Nottingham, UK.

## Abstract

Two colorectal (HT29, LoVo) and one gastric (MKN45) human tumour cell lines were examined for their in vitro trophic response to human gastrin-17. MKN45 and HT29 responded by increased 75Se selenomethionine uptake to exogenous gastrin (139 +/- 5.5% and 123 +/- 3% of control values respectively) whereas LoVo showed no significant response to this hormone. When these same cell lines were grown as xenografts in nude mice, similar responses were seen to exogenously administered human gastrin-17 (10 micrograms mouse-1 day-1, subcutaneous injection). MKN45 xenografts showed a greater response to continuously administered gastrin (osmotic mini-pumps, (10 micrograms mouse-1 day-1) when compared to the same dose given via a subcutaneous bolus injection. The hormone-treated xenografts had a two-fold increase in tumour cross-sectional area and growth rate when compared to saline-treated controls. Dose-response studies revealed that 0.4 micrograms gastrin mouse-1 day-1 appeared to be the minimally effective dose. As gastric and colorectal tumour cells show a trophic response to gastrin, antagonists of the gastrin receptor may prevent this effect causing tumour stasis. The gastric tumour cell line, MKN45, is gastrin-responsive and would be an ideal model for screening potent receptor antagonists.


Br. J. Cancer (1989), 59, 554–558 © The Macmillan Press Ltd., 1989

### Gastrin: growth enhancing effects on human gastric and colonic tumour cells

S. Watson[1], L. Durrant[1] & D. Morris[2]

[1]*Cancer Research Campaign Laboratories, University of Nottingham, Nottingham NG7 2RD, UK; and* [2]*Department of Surgery, Queens Medical Centre, Nottingham NG7 2UH, UK.*

**Summary** Two colorectal (HT29, LoVo) and one gastric (MKN45) human tumour cell lines were examined for their *in vitro* trophic response to human gastrin-17. MKN45 and HT29 responded by increased $^{75}$Se-selenomethionine uptake to exogenous gastrin ($139\pm5.5\%$ and $123\pm3\%$ of control values respectively) whereas LoVo showed no significant response to this hormone. When these same cell lines were grown as xenografts in nude mice, similar responses were seen to exogenously administered human gastrin-17 ($10\,\mu g\,mouse^{-1}\,day^{-1}$, subcutaneous injection). MKN45 xenografts showed a greater response to continuously administered gastrin (osmotic mini-pumps, ($10\,\mu g\,mouse^{-1}\,day^{-1}$) when compared to the same dose given via a subcutaneous bolus injection. The hormone-treated xenografts had a two-fold increase in tumour cross-sectional area and growth rate when compared to saline-treated controls. Dose–response studies revealed that $0.4\,\mu g$ gastrin $mouse^{-1}\,day^{-1}$ appeared to be the minimally effective dose. As gastric and colorectal tumour cells show a trophic response to gastrin, antagonists of the gastrin receptor may prevent this effect causing tumour stasis. The gastric tumour cell line, MKN45, is gastrin-responsive and would be an ideal model for screening potent receptor antagonists.

The hormonal control of breast and prostatic tumours has become well established (Carter *et al.*, 1977; Higgins & Hodges, 1941) and clinically effective hormone antagonists such as tamoxifen are widely used.

Gastrointestinal (GI) tract tumours are among the commonest found in humans (UK data: colon and rectum, 25,000; stomach 11,000 cancer deaths per year). Radiotherapy and cytotoxic drug therapy have failed to produce significant therapeutic results. An investigation into the hormonal control of such tumours with a view to developing antagonists of hormone action may yield alternative modes of treatment for gut tumours.

Gastrin appears to be an important trophic hormone for both normal and malignant GI mucosal cells. Gastric and duodenal mucosal cells were shown to respond to pentagastrin when cultured *in vitro* (Miller *et al.*, 1973; Lichtenberger *et al.*, 1973). Naturally occurring gastrins (G17I, G17II, G34II) were several times more potent than pentagastrin in their trophic effects on normal mucosa (Johnson, 1977). More recently it was shown that the density of fundic mucosal endocrine cells was positively related to levels of serum gastrin (Borch *et al.*, 1986).

GI tract tumour cells have been shown to respond to gastrin. Both established colorectal cell lines (Kusyk *et al.*, 1986; McRae *et al.*, 1986) and gastric cell lines (Watson *et al.*, 1988) respond to gastrin *in vitro*. The same has been shown with gastric and colorectal primary tumour cells (Moyer, 1983; Sirinek *et al.*, 1985).

The need to produce gastrin (receptor) antagonists and test them in reliable *in vitro* and *in vivo* systems is fundamental to determining the possible role of such agents in the treatment of GI cancer. This paper compares the gastrin responsiveness of human gastric and colorectal cell lines *in vitro* and *in vivo*.

### Materials and methods

### *Established cell lines*

Three established human gastric and colorectal cell lines were examined for gastrin-dependence: (i) MKN45 is a human adenocarcinoma cell line originally derived from a metastatic tumour of the liver from a 62-year-old Japanese woman with an undifferentiated adenocarcinoma of the stomach (Hojo, 1977); (ii) HT29 and LoVo are human colorectal adenocarcinoma cell lines.

The cells used for *in vitro* assays had previously been grown in immunocompromised (nude) mice (Olac, Oxfordshire) and had been passaged less than five times *in vitro*, as previously described (Watson *et al.*, 1988). The cells were grown *in vitro* in Dulbecco's growth medium (DMEM) supplemented with 10% fetal calf serum (FCS, Gibco) at 37°C in the presence of 5% $CO_2$.

### In vitro *assessment of cell growth in the presence of gastrin*

Cells were synchronised in the G1 phase of cell growth by the addition of excess thymidine and cell growth was assessed by $^{75}$Se-selenomethionine incorporation as previously described (Watson *et al.*, 1988). Human gastrin-17 (G17, Sigma, Dorset) concentrations between 0.02 and $10.0\,\mu g\,l^{-1}$ were used.

### *Growth of cell lines as xenografts in nude mice*

Xenografts were initiated by injection of cell lines as previously described (Watson *et al.*, 1988). The resultant tumours were aseptically dissected, mechanically minced and $5\,mm^3$ pieces of tumour tissue transplanted into the animals and randomised into experimental groups. The control groups of animals received phosphate-buffered saline (PBS) either by a subcutaneous bolus injection (0.2 ml) daily or by continuous infusion with the use of 14 day osmotic minipumps (Alzet, London, model 2002). Gastrin-treated animals received G17 either subcutaneously (single dose, $10\,\mu g\,mouse^{-1}\,day^{-1}$, 0.2 ml) or by 14 day mini-pump (0.4, 2.0, $10.0\,\mu g\,mouse^{-1}\,day^{-1}$).

Tumour growth was evident from between days 4 and 7. The tumours were measured two or three times a week, with the use of calipers, for 3 weeks and the measurements were made by an independent observer. The two largest perpendicular diameters of the tumours were measured and from these the cross-sectional areas were derived.

### *Measurements of serum gastrin levels after administration of gastrin*

Nude mice were starved overnight and then tail bled to measure fasting serum gastrin levels.

Aliquots of $200\,\mu l$ blood were removed per mouse at each tail bleed, which yielded $20\,\mu l$ serum. The gastrin levels of the serum were then assayed using a Radioimmunoassay kit (CIS Labs, UK) with the detection limit being $15\,pg\,ml^{-1}$.

Correspondence: S. Watson.
Received 25 May 1988, and in revised form, 30 November 1988.

*Statistics*

Statistical analysis on both *in vitro* and *in vivo* data was performed by using the paired Student's *t* test and the term '% of control' used to compare data is defined as

$$\frac{\text{c.p.m. in presence of gastrin}}{\text{c.p.m. in absence of gastrin}} \times 100$$

*In vivo* data were also subjected to a one-way analysis of variance which was performed over the complete time scale of each experiment. Where replicates were performed, the standard error of the mean was calculated.

### Results

The cell lines examined for their gastrin dependence had been passaged less than five times *in vitro* as the growth response of tumour cells to gastrin may be lost on prolonged *in vitro* passage (Watson *et al.*, 1988).

Figure 1 shows a typical experiment in which the gastric cell line, MKN45 and the colorectal cell line, HT29 both incorporated $^{75}$Se-selenomethionine to greater levels in the presence of G-17. Maximum responses of $139 \pm 5.5\%$ (MKN45) and $123 \pm 3\%$ (HT29) of control values were achieved at gastrin concentrations of 0.2 and $1.0\,\mu g\,l^{-1}$ respectively (Figure 1). The colorectal cell line, LoVo, failed to achieve a significant response to G17.

The same cell lines were grown as xenografts in nude mice. In all xenograft experiments, an arbitrary tumour area of $1.5\,cm^2$ has been designated to indicate satisfactory tumour growth from transplanted tissue. With MKN45 xenografts (Figure 2a) 1/9 of PBS-treated mice achieved a tumour cross-sectional area greater than $1.5\,cm^2$ whereas 4/10 gastrin treated mice had tumours of such a size. The mean growth rates of PBS-treated and gastrin-treated xenografts were $54 \pm 8\,mm^2\,day^{-1}$ and $68 \pm 11\,mm^2\,day^{-1}$ respectively. Statistical analysis revealed no significant difference between the two groups.

The growth of LoVo xenografts was not influenced by administration of gastrin (Figure 2b). HT29 xenografts had a greater mean growth rate in the absence of gastrin ($133 \pm 21\,mm^2\,day^{-1}$) when compared to MKN45 ($54 \pm 8\,mm^2\,day^{-1}$) and LoVo ($54 \pm 9\,mm^2\,day^{-1}$) ($P < 0.01$). In the PBS-treated animal group, 3/4 HT29 xenografts achieved a tumour size greater than $1.5\,cm^2$ compared to 5/5 in the gastrin-treated group, which had a mean growth rate of $147 \pm 12\,mm^2\,day^{-1}$. These differences, however, were not significant.

To try and develop a more gastrin-responsive xenograft line, the four MKN45 xenografts achieving an area of greater than $1.5\,cm^2$ in the gastrin-treated group (Figure 2a) were removed, the tissue was combined and retransplanted into two more groups of experimental animals. These were randomised into PBS and gastrin-treated ($10\,\mu g\,mouse^{-1}\,day^{-1}$) groups. From Figure 3, it can be seen that 3/10 PBS-treated mice had xenografts greater than $1.5\,cm^2$ compared to 7/10 gastrin-treated animals. This was found to be statistically significant (one way analysis of variance, $P < 0.01$). The mean growth rate of PBS-treated xenografts was $68 \pm 9\,mm^2\,day^{-1}$ compared to $90 \pm 9\,mm^2\,day^{-1}$ in the gastrin treated tumours.

As human gastrin-17 has a short half-life in the circulation (6 min; Walsh & Grossman, 1975), it was decided to administer the hormone by continuous infusion with the use of an osmotic mini-pump. Gastrin-17 was delivered over a 14 day period from the day of xenograft transplantation. Figure 4 shows the growth response of both MKN45 and HT29 xenografts to pumped gastrin. The growth rate of MKN45 xenografts in response to gastrin ($10\,\mu g\,mouse^{-1}\,day^{-1}$) was $178 \pm 20\,mm^2\,day^{-1}$ compared to $94 \pm 9\,mm^2\,day^{-1}$ in the PBS treated controls ($P < 0.01$, Student's *t* test, $P < 0.01$, one-way analysis of variance over entire experiment).

**Figure 1** The growth response to G17 of established human gastric (**a**) (MKN45) and colorectal; (**b**) HT29; (**c**) LoVo cell lines. Points, means of triplicate samples, $^*P < 0.0001$, $^{**}P < 0.01$. Student's *t* test.

The growth rate of HT29 xenografts in response to constantly infused gastrin was $148 \pm 21\,mm^2\,day^{-1}$ compared to $105 \pm 15\,mm^2\,day^{-1}$, in the PBS-treated controls. There was a trend to increased growth in the presence of gastrin which was not significant.

A titration was performed on the growth response of MKN45 xenografts to pumped gastrin. Gastrin was administered at decreasing concentrations of 10.0, 2.0 and $0.4\,\mu g\,mouse^{-1}\,day^{-1}$ and the growth of the corresponding xenografts was measured over a 24-day period (Figure 5). For comparative purposes, the mean of the tumour measurements for each group was calculated.

By day 19, animals treated with the two higher gastrin concentrations had xenografts of greater size than the control animals ($10\,\mu g\,mouse^{-1}\,day^{-1}$; $P < 0.05$,

**Figure 2** The growth response of human gastric (**a**) (MKN45) and colorectal; (**b**) HT29; (**c**) LoVo tumour xenografts to subcutaneously administered gastrin ($10\,\mu g$ mouse$^{-1}$ day$^{-1}$). Individual tumour measurements: i, PBS control ●; ii, gastrin-treated ○.

$2\,\mu g$ mouse$^{-1}$ day$^{-1}$; $P<0.01$). At day 21 the xenografts of mice treated with all three gastrin concentrations (10, 2, $0.4\,\mu g$ mouse$^{-1}$ day$^{-1}$) were significantly greater than the control ($P<0.05$, $P<0.01$, $P<0.05$ respectively). By day 24, xenografts of animals treated with the lower gastrin concentrations were not significantly different from the control, whilst xenografts of animals treated with 10 and $2\,\mu g$ mouse$^{-1}$ day$^{-1}$ remained so ($P<0.05$, $P<0.01$).

Measurements of fasting serum gastrin levels were taken during each dose of administered gastrin. In PBS-treated animals a mean fasting serum gastrin concentration of $134 \pm 34$ pg ml$^{-1}$ was achieved whereas with pumped gastrin at 10, 2 and $0.4\,\mu g$ mouse$^{-1}$ day$^{-1}$, mean fasting serum

**Figure 3** The growth of gastrin-responsive MKN45 xenografts in nude mice: response to subcutaneously administered gastrin. Individual tumour measurements: i, PBS control ●; ii, gastrin-treated ○ ($10\,\mu g$ mouse$^{-1}$ day$^{-1}$). *$P<0.01$, one-way analysis of variance.

gastrin levels of $307 \pm 100$, $181 \pm 45$ and $155 \pm 34$ pg ml$^{-1}$ were achieved ($n=5$).

### Discussion

As radiotherapy and chemotherapy of GI tumours have failed to produce beneficial therapeutic results an alternative mode of treatment has been sought. This study investigates the hormonal control of such tumours with a view to developing antagonists, if tumour growth shows hormonal dependence.

Early results showed that GI tract tumour cells have a low growth response to gastrin after long-term *in vitro* growth (Watson *et al.*, 1988). However, if the same cells were grown as xenografts in nude mice and re-examined after short-term *in vitro* growth, they showed an elevated response.

In the present study, MKN45 and HT29 cells incorporated elevated levels of $^{75}$Se-selenomethionine in the presence of human G-17 whereas LoVo cells failed to respond.

**Figure 4** The growth response of MKN45 (**a**) and HT29 (**b**) xenografts to gastrin administered by continuous infusion (via an osmotic mini-pump, 10 μg mouse$^{-1}$ day$^{-1}$). Individual tumour measurements: i, PBS control ●; ii, gastrin treated ○. *$P<0.01$, one-way analysis of variance.

**Figure 5** The dose–response of MKN45 xenografts to gastrin administered via an osmotic mini-pump. Mean tumour measurements ($n=5$), PBS control □, gastrin (μg mouse$^{-1}$ day$^{-1}$) 0.4 ▨, 2.0 ▩, 10.0 ▤.

These same cell lines, grown as xenografts in nude mice, did not respond significantly to daily subcutaneous injections of human G-17. This is not surprising as the tumour cells will only be exposed to the hormone for a short time, as the serum half-life of G-17 is 6 min (Walsh & Grossman, 1975). However, when MKN45 xenografts were exposed to continuously administered gastrin it induced a two-fold increase in cross-sectional area and mean rate of tumour growth when compared to saline-treated controls. HT29 xenografts showed a trend to increased growth in the presence of continuously infused gastrin that was not statistically significant.

In the MKN45 xenograft model, pumped gastrin which induced up to three fold increases in circulating fasting serum gastrin levels (when compared to PBS-treated animals) appeared to increase the growth of the tumours in the short-term. In accord with this, studies in humans have revealed that fasting serum gastrin levels are elevated 8.5-fold in patients with colorectal cancer (Smith *et al.*, 1987) and 3.5-fold in patients with carcinoma of the stomach (McGuigan & Trudeau, 1973).

A similar study was performed with xenotransplanted human gastric and colon carcinomas (Sumiyoshi *et al.*, (1984). A human gastric carcinoma, SC-6-JCK, responded trophically to gastrin whereas a second human gastric carcinoma, ST15, and a colonic adenocarcinoma, Co3, failed to respond. However, the response with SC-6-JCK was obtained with pentagastrin (10 μg mouse$^{-1}$ day$^{-1}$) which is known to be less potent at promoting a trophic response with normal cells than gastrin-17 (Johnson, 1977) and has been shown to have a lower binding affinity for the gastrin receptor when compared to gastrin-17 (P. Singh, personal communication). The pentagastrin was also administered via a single subcutaneous injection. It is possible therefore that SC-6-JCK may have greater sensitivity to the action of gastrin than MKN45.

A recent study has shown that a mouse colon adenocarcinoma cell line, MC26, responded to pentagastrin (3.25–15.00 μg mouse$^{-1}$ day$^{-1}$) administered by intraperitoneal injections (Winsett *et al.*, 1986). It has also been found that both exogenous gastrin (pentagastrin, 2 mg kg$^{-1}$, subcutaneous injection) and endogenous hypergastrinaemia (induced by antral exclusion) exerted trophic effects on chemically induced colorectal neoplasms in the rat (McGregor *et al.*, 1982). The lack of gastrin response seen in the human colorectal tumour cells examined in the present study may be dose related and further experiments will be performed to investigate this.

In conclusion, we have demonstrated that a human gastric adenocarcinoma cell line, MKN45, when initiated as a xenograft in nude mice, almost doubles in size upon continuous administration of human G-17. Together with the cell line's gastrin responsiveness *in vitro* it is an ideal candidate for screening gastrin (receptor) antagonists with a view to examining them as potential therapeutic agents.

These studies were supported by ICI, Macclesfield and the Cancer Research Campaign, UK. We are grateful to E.A. Donaghue and J. Haynes for excellent technical assistance. We would like to thank Professor Motoyama for the use of the human gastric adenocarcinoma cell line, MKN45.

### References

BORCH, K., RENVALL, H., LIEDBERG, G. & ANDERSEN, B.N. (1986). Relations between circulating gastrin and endocrine cell proliferation in the atrophic gastric fundic mucosa. *J. Gastroent.*, **21**, 357.

CARTER, A.C., SEDRANSK, N., KELLEY, R.M. & 4 others (1977). Diethylstibestiol recommended dosages for different categories of breast cancer patients. *JAMA*, **237**, 2079.

HIGGINS, C. & HODGES, C.O. (1941). Studies on prostatic cancer. 1. The effect of castration, of estrogen and of androgen injection on serum phosphatases in metastatic carcinoma of the prostate. *Cancer Res.*, **1**, 293.

HOJO, J. (1977). Establishment of cultured cell lines of human stomach cancer origin and their morphological characteristics. *Niigata Igakukai Zasshi*, **91**, 737.

JOHNSON, L.R. (1979). New aspects of the trophic action of gastrointestinal hormones. *Gastroenterology*, **72**, 788.

KUSYK, C.J., McNIEL, N.O. & JOHNSON, L.R. (1986). Stimulation of growth of a colon cancer cell line by gastrin. *Am. J. Physiol.*, **251**, 597.

LICHTENBERGER, L., MILLER, L.R., ERWIN, D.N. & JOHNSON, L.R. (1973). Effect of pentagastrin on adult rat duodenal cells in culture. *Gastroenterology*, **65**, 242.

McGREGOR, D.B., JONES, R.D., KARLIN, D.A. & ROMSDAHL, M.M. (1982). Trophic effects of gastrin on colorectal neoplasms in the rat. *Ann. Surg.*, **195**, 219.

McGUIGAN, J.E. & TRUDEAU, W.L. (1973). Serum and tissue gastrin concentrations in patients with carcinoma of the stomach. *Gastroenterology*, **64**, 22.

McRAE, L.J., KIENER, P.A. & CATINO, J.J. (1986). Role of gastrin and gastrin receptors in the growth of human colon carcinoma cells. *J. Cell. Biol.*, **103**, 22a.

MILLER, L.R., JACOBSON, E.D. & JOHNSON, L.R. (1973). Effect of pentagastrin on gastric mucosal cells grown in tissue culture. *Gastroenterology*, **63**, 254.

MOYER, M.P. (1983). A rapid reproducible method for processing human solid tumours for *in vitro* culture. *J. Tissue Culture Meth.*, **8**, 63.

SIRINEK, K.R., LEVINE, H.A. & MOYER, M.P. (1985). Pentagastrin stimulates *in vitro* growth of normal and malignant human colon epithelial cells. *Am. J. Surg.*, **149**, 35.

SMITH, J.P., WOOD, J.G. & SOLOMON, T.E. (1987). Elevated gastrin levels in patients with colorectal cancer and adenomatous polyps. *Gastroenterology*, **92**, 1646.

SUMIYOSHI, H., YASUI, W., OCHIAI, A. & TAHARA, E. (1984). Effects of gastrin on tumour growth and cyclic nucleotide metabolism in xenotransplantable human gastric and colonic carcinomas in nude mice. *Cancer Res.*, **44**, 4276.

WALSH, J.H. & GROSSMAN, M.I. (1975). Gastrin. *N. Engl. J. Med.*, **292**, 1324.

WATSON, S.A., DURRANT, L.G. & MORRIS, D.L. (1988). Growth-promoting action of gastrin on human colonic and gastric tumour cells cultured *in vitro*. *Br. J. Surg.*, **75**, 342.

WINSETT, O.E., TOWNSEND, C.M., GLASS, E.J. & THOMPSON, J.C. (1986). Gastrin stimulates growth of colon cancer. *Surgery*, **99**, 302.